# Higher daytime systolic BP, prepregnancy BMI and an elevated sFlt-1/PlGF ratio predict the development of hypertension in normotensive pregnant women

**DOI:** 10.1186/s12958-022-01050-w

**Published:** 2022-12-23

**Authors:** Almudena Lara-Barea, Begoña Sánchez-Lechuga, Manuel Aguilar-Diosdado, Cristina López-Tinoco

**Affiliations:** 1 Department of Endocrinology and Nutrition, Puerta del Mar Hospital, 11009 Cádiz, Spain; 2 Biomedical Research and Innovation Institute of Cádiz (INiBICA), Puerta del Mar Hospital, 11009 Cádiz, Spain; 3 Department of Medicine, Cadiz University (UCA), 11003 Cádiz, Spain

**Keywords:** Ambulatory blood pressure monitoring, Predictor, Hypertensive disorders of pregnancy, Cytokine profile; sFlt-1/PIGF ratio

## Abstract

**Background:**

The risk of hypertensive disorders of pregnancy (HDP) varies in women with gestational diabetes mellitus (GDM), depending on the degree of insulin resistance and is also influenced by obesity. The aim of this study was to evaluate clinical features, blood pressure (BP) profiles and inflammatory markers, to identify patients with an elevated risk of developing HDP.

**Methods:**

A total of 146 normotensive pregnant women were studied. We analysed the relationships of BP profiles detected by ambulatory blood pressure monitoring (ABPM) with serum biomarkers and angiogenic factors and their association with the development of HDP.

**Results:**

Fourteen (9.6%) women developed HDP, of which 11 had GDM and 8 had obesity. Women with HDP had higher values of 24-h and daytime systolic/diastolic BP (113/69 vs. 104/64; 115/72 vs. 106/66 mmHg, respectively; *p* <  0.05). Higher levels of leptin (10.97 ± 0.82 vs. 10.2 ± 1.11; *p* = 0.018) andmonocyte chemoattractant protein-1 (MCP-1) (5.24 ± 0.60 vs. 4.9 ± 0.55; *p* = 0.044) and a higher soluble fms-like tyrosine kinase-1/placental growth factor (sFlt-1/PlGF) ratio (4.37 ± 2.2 vs. 2.2 ± 1.43; *p* = 0.003) were also observed in the HDP patients. Multivariate analysis showed that a higher sFlt-1/PlGF ratio was associated with an increased risk of developing HDP [OR = 2.02; IC 95%: 1.35–3.05]. Furthermore, higher daytime systolic BP [OR = 1.27; IC 95% 1.00–1.26] and prepregnancy body mass index (BMI) [OR = 1.14; IC 95%: 1.01–1.30] significantly increased the risk of developing HDP.

**Conclusions:**

Higher daytime systolic BP values, prepregnancy BMI and the sFlt-1/PlGF ratio are useful for identifying normotensive pregnant women with an increased risk of developing HDP.

## Background

Gestational diabetes mellitus (GDM) and hypertensive disorders of pregnancy (HDP) are two pathologies that most frequently complicate pregnancies, and imply an increase in maternal and neonatal morbidity [1, 2] and an increased risk of developing cardiovascular disease [1, 3, 4]. Both conditions share risk factors and are related to each other, as HDP (as gestational hypertension and preeclampsia) is 1.5–2 times more common in women with GDM [5, 6], and an increased risk of developing GDM has been described in women with history of preeclampsia in previous pregnancies [7].

Several studies have proposed that both clinical conditions share – at least partly – some pathophysiological mechanisms in which insulin resistance may play a key role [8]. In addition, an increased proinflammatory state associated with HDP [9, 10] and GDM [11, 12] has been described, and in that respect, our group has observed an angiogenic imbalance, characterized by an increased soluble fms-like tyrosine kinase–1/placental growth factor (sFlt-1/PlGF) ratio, as a valid predictor of the development of HDP in women with GDM, as well as its association with obstetric and perinatal complications [13].

Ambulatory blood pressure monitoring (ABPM) provides a larger number of blood pressure (BP) measurements, allows assessment of the circadian rhythm and detects BP alterations that correlate with target organ involvement and cardiovascular morbidity and mortality in the general population [14]. In pregnancy, previous works have supported the use of ABPM to detect subclinical changes in BP patterns (BP changes/alterations that can only be detected by ABPM) in pregnant women who are at higher risk of developing HDP [15–17]. Importantly, we recently published a study reporting that high nocturnal systolic BP levels are related to the development of HDP in pregnant women with GDM and obesity [18]. In addition, these alterations were found to be related to poor obstetric and perinatal outcomes.

While the interaction between endothelial damage, insulin resistance, and the development of HDP has been reported [19–21], the complex relationship between proinflammatory cytokines and subclinical BP alterations in pregnant women with GDM has not been demonstrated. The aim of this study was to evaluate the relationship between inflammatory markers and BP profiles as measured by ABPM, in normotensive women with and without GDM to identify those with an elevated risk of developing HDP.

## Methods

A prospective observational study was performed by recruiting normotensive pregnant women who were selected consecutively from the Endocrinologist and Obstetric clinic of the Puerta del Mar University Hospital (Cádiz, Spain). The inclusion criteria were as follows: women with singleton physiological pregnancy and normal BP at the time of enrolement (ambulatory systolic BP ≤ 130 mmHg and diastolic BP ≤ 80 mmHg). The exclusion criteria were as follows: women with chronic hypertension or receiving antihypertension medication; a diagnosis of pregestational diabetes, morbid obesity (defined by body mass index (BMI) ≥ 40 kg/m^2^), or placental insufficiency, and the presence of concomitant systemic disease or smoking.

### Procedure

The study was approved by the Hospital Research Ethics Board of Puerta del Mar Hospital (code number 1507-N-16) following the guidelines of the Declaration of Helsinki. Written informed consent was obtained from all participants.

At the time of inclusion in the study, maternal clinical data were recorded: family history of hypertension, age, pregestational weight and BMI, presence of obesity (defined as having a prepregnancy BMI ≥ 30 kg/m^2^), diagnosis of GDM, obstetric history, parity and history of HDP or GDM in a previous pregnancy. Pregnant women at high risk of preeclampsia were treated with 100 mg of aspirin daily from 12 until 37 weeks according to our hospital protocol based on NICE guidelines [22]: presence of any high-risk factors, including: hypertensive disease during a previous pregnancy, chronic kidney disease, autoimmune disease such as systemic lupus erythematosus or antiphospholipid syndrome, type 1 or type 2 diabetes and chronic hypertension, or presence of more than one moderate risk factor, including first pregnancy, age of 40 years or older, pregnancy interval of more than 10 years, BMI > 35 kg/m^2^ at first visit, family history of preeclampsia, and multi-fetal pregnancy. The diagnosis of GDM was established using a two-step approach according to the criteria of the National Diabetes Data Group [23]: in all pregnant women between 24 and 28 weeks of gestation a screening test was performed with a 50 g glucose test. Women with a positive screening test (1-h blood glucose > 7.8 mmol/L) underwent a confirmatory 3-h, 100 g oral glucose tolerance test (OGTT). GDM was diagnosed with abnormally high values of two of the following thresholds: fasting glucose, 5.8 mmol/L; 1-h, 10.5 mmol/L; 2-h, 9.1 mmol/L; 3-h, 8.0 mmol. All women with GDM received complex dietary counselling at diagnosis, which consisted of a high-protein diet (at least 1.1 g protein/kg per day) with a caloric intake between 25 and 35 kcal/kg of b.w. per day adjusted for pregestational BMI. If glucose targets were not achieved (fasting glucose < 5.3 mmol/L and postprandial glucose at 1 h < 7.8 mmol/L), insulin therapy was initiated.

During the third trimester of pregnancy, pregnant women were scheduled for a first and single visit between 28 and 32 weeks at 08:30–09:30. An interview and physical examination were carried out, fasting blood samples were collected, BP was measured and 24-h ABPM was performed. After delivery, obstetric and perinatal data were retrospectively reviewed.

### Blood pressure measurements

The conventional office BP was measured on the nondominant arm with an automated BP monitor (Omron HEM-7200-E (Kyoto, Japan)) in a sitting position. The measurement was performed twice on the same day and before ABPM was undertaken. For the 24-h recordings, the Spacelabs 90,207 monitor (Spacelabs, Redmond,WA, USA) was used. The monitor was programmed to perform the measurements every 20 min during the day and every 30 min at night; daytime hours were set as the period between 6.00 and 22.00 h, and night-time hours were set from 22.00 to 6.00. Having completed a diary of activities for the night-time rest, the actual sleep time was corrected for each patient. ABPM values with at least 66% successful measurements and at least one record per hour were considered valid. The following ABPM circadian patterns were established: dipper pattern (defined by a nocturnal BP reduction between 10 and 20% compared to the daytime period), extreme dipper pattern (nocturnal BP reduction of 20% or more), nondipper pattern (BP decrease of less than 10% in the nocturnal period compared to the daytime period) and riser pattern (mean nocturnal BP increase relative to the daytime period). The last three patterns were consideredpathological circadian patterns.

### Laboratory measurements

Specimen blood samples were collected for biochemical analysis (including glucose, HbA1c, uric acid level, HOMA index and lipid profile), following a minimum of 8 h fast. The blood sample was centrifuged (7 min; 3000 rpm) and aliquots were stored at − 80 °C in a freezer until the batch measurement of cytokines. Cytokine levels (including sFlt-1, PlGF, adiponectin, leptin, MCP-1, PAI-1, resistin, NGF, TNFα, HGF, and FGF-2) were measured in maternal plasma using commercial kits following the manufacturer’s instructions (Millipore, Billerica, MA, USA) that uses the xMAP technology (Luminex Corporation, Austin, TX, USA). Levels of cytokines were logarithmically transformed due to large values.

### Pregnancy outcomes

After delivery, the following obstetric and perinatal data were retrospectively reviewed: maternal weight gain, gestational age at delivery, route of delivery, delivery complications, birthweight and customized percentile, and the Apgar score of the newborn. Intrauterine growth restriction (IUGR) was defined as a birthweight less than the 5th percentile on a customized pediatric curve; small for gestational age (SGA) was defined as a birthweight below the 10th percentile, and macrosomia was designated as a birthweight above the 95th percentile for gestational age. We considered the presence of HDP, including gestational hypertension (BP > 140/90 mmHg in a woman who was normotensive before the 20th week of gestation and whose BP returned to normal by 12 weeks after delivery) and preeclampsia (defined as the new onset of hypertension after the 20th week of gestation in a previously normotensive woman, who developed proteinuria or end-organ dysfunction [24]).

### Statistical analysis

Statistical analyses carried out using the IBM SPSS program (version 24.0 software for MS Windows). The normality of the variables was assessed with the Shapiro–Wilk test. Continuous variables were reported as the mean and standard deviation (SD) and were compared between independent groups using Student’s *t* –test or the Mann–Whitney *U* –test for nonparametric variables. Categorical variables were expressed as frequencies and percentages and were compared using the χ2 test or Fisher’s exact test as appropriate. The correlation between two variables was studied with the Pearson test or Spearman’s correlation coefficient. *p* values less than 0.05 were defined as significant in all two-tailed analyses. Multivariate analysis was performed using nonconditional logistic regression. The stepwise technique was used to select the independent variables introduced into the model based on clinical and statistical criteria of *p* <  0.05 in a bivariate analysis. The fit of the final model was tested using the Hosmer–Lemeshow test.

## Results

**Fig. 1:**
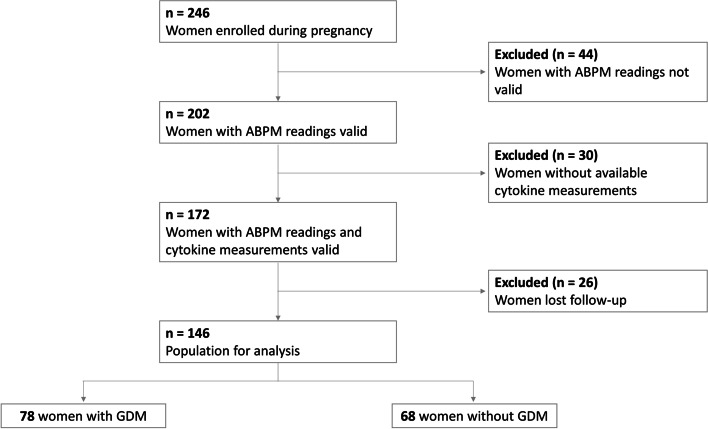
Study flowchart. ABPM: ambulatory blood pressure monitoring; GDM: gestational diabetes mellitus

A total of 246 normotensive pregnant women were enrolled. We excluded women with < 66% of valid ABPM readings and nonavailability of cytokine measurements and women who gave birth elsewhere (Fig. 1). Finally, 146 pregnant women with normal BP were included in the analysis, 78 patients diagnosed with GDM and 68 with normal glucose tolerance. Women with GDM were found to be older than non-GDM women (34.3 ± 3.6 vs. 32.8 ± 4.8; *p* = 0.029) and, as expected, had significantly higher triglycerides (2.3 ± 0.9 vs. 2.0 ± 0.6 mmol/L; *p* = 0.04), basal glucose (4.9 ± 0.6 vs. 4.6 ± 0.3 mmol/L; *p* = 0.018) and HbA1c levels (5 ± 0.4 vs. 4.8 ± 0.3%; *p* = 0.003). The rest of the studied variables showed no statistically significant differences between the groups.

Fourteen patients (9.6%) developed HDP; 10 presented with gestational hypertension, and four developed added preeclampsia. The demographic and clinical characteristics of women who developed some type of HDP and normotensive women are summarized in Table 1. No differences in maternal age, family history of hypertension, obstetric history, parity or gestational age at the time of inclusion were observed. Of the 14 hypertensive pregnant women, eight had obesity (57.16%) and 11 had GDM (78.6%), these included the four patients who developed preeclampsia. Hence, pregnant women with HDP showed significantly greater prepregnancy BMI and higher HbA1c and basal glucose levels, without statistically significant differences in the rest of the laboratory variables measured (Table 1). We also found that office systolic and diastolic BP (measured at 28–32 weeks of pregnancy) were significantly higher in women who subsequently developed HDP, and the rate of aspirin prophylaxis was higher in this group.

**Table 1:** Baseline clinical characteristics and laboratory variables in women with and without HDP

Variable	HDP (*n* = 14)	Non-HDP (*n* = 132)	*p* Value
*Clinical characteristics*			
Maternal age (y) *	33.6 ± 4.4	33.6 ± 4.3	0.9
Family history AHT ^†^	4 (28.6%)	58 (43.9%)	0.2
Parity *	1.71 ± 1.4	1.89 ± 0.9	0.5
Previous history of GDM ^†^	1 (7.1%)	19 (14.4%)	0.4
Previous history of preeclampsia ^†^	1 (7.1%)	2 (1.5%)	0.1
Gestational age at enrollement (w) *	31.2 ± 1.9	31.1 ± 1.9	0.8
Pregestational BMI (kg/m^2^) *	30.1 ± 6.8	26.0 ± 5.0	0.046
Obesity ^†^	8 (57.1%)	28 (21.2%)	0.006
GDM ^†^	11 (78.6%)	67 (50.2%)	0.047
Insulin treatment ^†^	5 (45.4%)	24 (35.8%)	0.2
Office Systolic BP (mmHg) *	118.5 ± 15.7	109.7 ± 15.4	0.043
Office Diastolic BP (mmHg) *	74.5 ± 9.7	65.9 ± 9.0	0.001
ASA prophylaxis ^†^	5 (35.7%)	14 (10.6%)	0.008
*Laboratory parameters*			
Basal glucose (mmol/L) *	4.9 ± 0.5	4.8 ± 0.5	0.039
HbA1c (%)*	5.2 ± 0.5	4.8 ± 0.3	0.002
HOMA-IR *	2.9 ± 1.1	2.0 ± 1.3	0.09
Albumin/creatinine (mg/g) *	28.3 ± 60.0	6.3 ± 6.6	0.1
Uric acid (mmol/L) *	0.24 ± 0.06	0.22 ± 0.13	0.6
Total-cholesterol (mmol/L) *	6.52 ± 0.86	6.45 ± 1.19	0.8
LDL-cholesterol (mmol/L) *	3.76 ± 0.8	3.66 ± 1.22	0.7
HDL-cholesterol (mmol/L) *	1.99 ± 0.42	1.92 ± 0.47	0.6
Triglycerides (mmol/L) *	2.2 ± 0.6	2.1 ± 0.8	0.6

* Data expressed as means ± standard deviation; comparisons between different groups were done by the Student’s t– test for parametric variables and the Mann–Whitney U– test for nonparametric variables

^†^ Data expressed as *n* (%); comparisons among groups were done using the χ2 test, or Fisher’s exact test for nonparametric contrasting

*HDP* hypertensive disorders of pregnancy, *AHT* arterial hypertension, *BMI* body mass index, *GDM* gestational diabetes mellitus, *BP* blood pressure, *ASA* acetylsalicylic acid, *HbA1c* glycated hemoglobin, *LDL* low-density lipoprotein, *HDL* high-density lipoprotein

With regard to obstetric and perinatal outcomes, hypertensive women delivered earlier and the difference in gestational age at delivery was almost significant; however, preterm delivery was significantly more common in this group. On the other hand, birthweight and customized percentile were significantly lower in women who developed HDP than in those who did not, and a higher rate of SGA and IUGR was observed in these patients, as shown in Table 2. Regarding neonatal complications, the rate of hypoglycemia was significantly higher in women who developed HDP, without significant differences in the remaining complications analysed.

**Table 2:** Obstetric and perinatal outcomes in women with and without HDP

Variable	HDP (*n* = 14)	Non-HDP (*n* = 132)	*p* Value
*Obstetric and perinatal outcomes*			
Weight gain (kg) *	10.9 ± 5.0	8.8 ± 4.2	0.1
Gestational age at delivery; (wk) *	38.4 ± 1.8	39.4 ± 1.1	0.06
Preterm delivery (< 37 wk)^†^	3 (21.4%)	0	< 0.001
Instrumental delivery ^†^	5 (35.7%)	30 (22.7%)	0.2
Cesarean section ^†^	5 (35.7%)	34 (25.8%)	0.3
Birthweight (g) *	2901 ± 667	3318 ± 472	0.038
Customized percentile *	28.6 ± 29.8	47.1 ± 29.1	0.026
*Neonatal complications* ^†^			
Macrosomia	0	16 (12.1%)	0.1
SGA	6 (42.9%)	16 (12.1%)	0.008
IUGR	4 (28.6%)	7 (5.3%)	0.012
Hypoglycemia	3 (21.4%)	4 (3%)	0.002
Hyperbilirubinemia	1 (7.1%)	6 (4.5%)	0.6
Congenital malformations	1 (7.1%)	1 (0.8)	0.051
Admission to the neonatal ICU	1 (7.1%)	5 (3.8%)	0.5

* Data expressed as means ± standard deviation; comparisons between different groups were done by the Student’s t– test for parametric variables and the Mann–Whitney U– test for nonparametric variables

^†^ Data expressed as *n* (%); comparisons among groups were done using the χ2 test, or Fisher’s exact test for nonparametric contrasting

*SGA* small for gestational age, *IUGR* intrauterine growth restriction

By analysing the relationship between cytokine and angiogenic factor concentrations and the development of HDP, we observed significantly lower maternal plasma PlGF levels in women with HDP than in normotensive women. MCP-1 and leptin levels, as well as the sFlt-1/PlGF ratio, were significantly higher in women who developed HDP (Table 3). No statistically significant differences were found in the rest of the measured markers.

**Table 3:** Bivariate analysis of the association between the development of HDP, biomarkers’ levels and ABPM parameters

Variable	HDP (*n* = 14)	Non-HDP (*n* = 132)	*p* Value
*Cytokines and biomarkers levels* *			
Adiponectin, (pg/ml)	10.22 ± 2.54	13.08 ± 2.93	0.06
Resistin (pg/ml)	7.43 ± 3.82	8.28 ± 3.28	0.37
PAI-1 (pg/ml)	8.04 ± 4.08	8.69 ± 3.48	0.51
NGF (pg/ml)	0.62 ± 0.84	0.73 ± 0.9	0.67
Leptin (pg/ml)	10.97 ± 0.82	10.2 ± 1.11	0.018
HGF (pg/ml)	6.65 ± 1.07	7.03 ± 1.23	0.41
MCP-1 (pg/ml)	5.24 ± 0.60	4.9 ± 0.55	0.044
TNFα (pg/ml)	0.35 ± 2.02	0.59 ± 1.13	0.49
FGF-2 (pg/ml)	3.8 ± 0.62	4.02 ± 0.63	0.22
sFlt-1 (pg/ml)	7.56 ± 0.93	7.25 ± 0.97	0.26
PIGF (pg/ml)	3.18 ± 1.79	5.1 ± 1.12	0.002
sFlt-1/PlGF ratio	4.37 ± 2.2	2.2 ± 1.43	0.003
*BP parameters*			
24 h SBP (mmHg) *	113.1 ± 14.4	104.2 ± 7.9	0.04
24 h DBP (mmHg) *	69.7 ± 9.2	64.1 ± 5.6	0.04
Daytime SBP (mmHg) *	115.7 ± 13.4	106.7 ± 8.6	0.001
Daytime DBP (mmHg) *	72.3 ± 8.2	66.6 ± 6.0	0.002
Nocturnal SBP (mmHg) *	107.5 ± 17.5	98.7 ± 7.8	0.08
Nocturnal DBP (mmHg) *	63.5 ± 12.1	58.5 ± 5.6	0.15
Pathological circadian pattern ^†^	9 (64.3%)	62 (46.9%)	0.17
Riser pattern ^†^	2 (14.3%)	6 (4.5%)	0.13
Nondipper pattern ^†^	4 (28.6%)	44 (33.3%)	0.72
Extrem dipper pattern	3 (21.4%)	12 (9.1%)	0.15
Dipper pattern ^†^	5 (35.7%)	70 (53%)	0.22

* Data expressed as means ± standard deviation; Comparisons between different groups were done by the Student’s t– test for parametric variables and the Mann–Whitney U– test for nonparametric contrasting

^†^ Data expressed as *n* (%); comparison among groups were done using the Fisher’s exact test

*HDP* hypertensive disorders of pregnancy, *PAI-1* plasminogen activator inhibitor-1, *NGF* nerve growth factor, *HGF* hepatocyte growth factor, *MCP-1* Monocyte Chemoattractant Protein-1, *TNFα* tumor necrosis factor alpha, *FGF-2* fibroblast growth factor-2, *sFlt-1* soluble fms-like tyrosine kinase-1, *PlGF* placental growth factor, *PB* blood pressure, *SBP* systolic blood pressure, *DBP* diastolic blood pressure

**Fig. 2:**
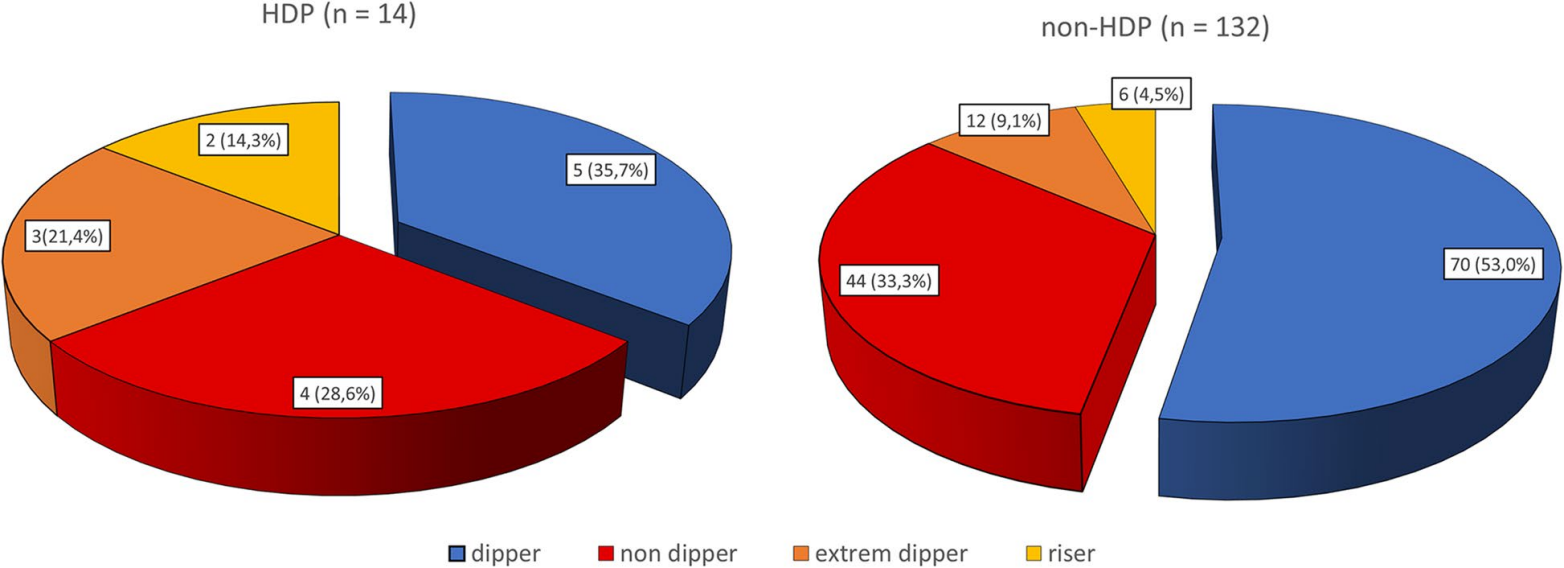
Prevalence of circadian patters in women with and without HDP. HDP: hypertensive disorders of pregnancy; extreme dipper pattern: subjects with a > 20% fall in nocturnal blood pressure; dipper pattern: subjects with a 10–20% fall in nocturnal blood pressure; non-dipper pattern: subjects with a 0–10% fall in nocturnal blood pressure; riser pattern: subjects with nocturnal blood pressure higher than diurnal blood pressure

Regarding ABPM parameters, significantly higher 24-hour and daytime systolic and diastolic BP levels were detected by ABPM between 28 and 32 weeks in patients who subsequently developed HDP compared to those who remained normotensive, (Table 3). Furthermore, the rate of pathological circadian patterns (including nondipper, extreme dipper and riser patterns) was higher in hypertensive women (64.3% vs. 46.9%; *p* = 0.17), but the difference did not reach statistical significance (Fig. 2). Conversely, we found that women with a pathological circadian pattern had significantly higher concentrations of triglycerides (2.3 ± 0.9 vs. 2.0 ± 0.6; *p* = 0.05) and sFlt-1/PlGF ratio (2.77 ± 1.7 vs. 2.07 ± 1.5; *p* = 0.01) than those who presented a dipper pattern. In addition, a lower customized percentile was also observed in patients without a dipper pattern relative to those with a dipper pattern (39.3 ± 27.2 vs. 51.1 ± 30.7; *p* = 0.017). No differences were found in the remainder of variables analysed.

**Fig. 3:**
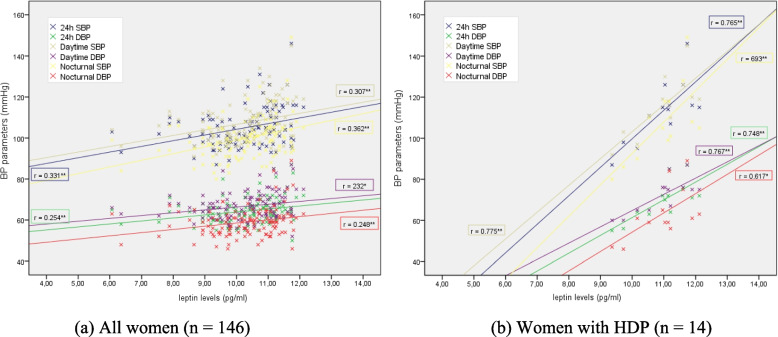
Correlation between ABPM and leptin levels in whole population (**a**); in women with HDP (**b**). * *p* < 0.05; ** *p* < 0.01. HDP: hypertensive disorders of pregnancy; PB: blood pressure; SBP: systolic blood pressure; DBP: diastolic blood pressure

There were moderate positive correlations between the levels of leptin and BP parameters detected by ABPM (Fig. 3a), but this correlation was stronger among the women who developed HDP (Fig. 3b): systolic (r: 0.765; *p* = 0.002) and diastolic BP (r: 0.748; *p* = 0.003) over 24 h, systolic (r: 0.775; *p* = 0.002) and diastolic BP (r: 0.767; *p* = 0.002) in the daytime period, and systolic (r: 0.693; *p* = 0.009) and diastolic BP (r: 0.617; *p* = 0.025) in the nocturnal period. Regarding the remaining cytokines measured, which were low but statistically significant (*p* < 0.05), positive correlations were found between MCP–1 levels and 24-h and nocturnal systolic BP and 24-h, daytime and nocturnal diastolic BP. In the group of women who had HDP, this correlation was significantly stronger for nocturnal systolic BP (*r* = 0.576; *p* = 0.039).

**Fig. 4:**
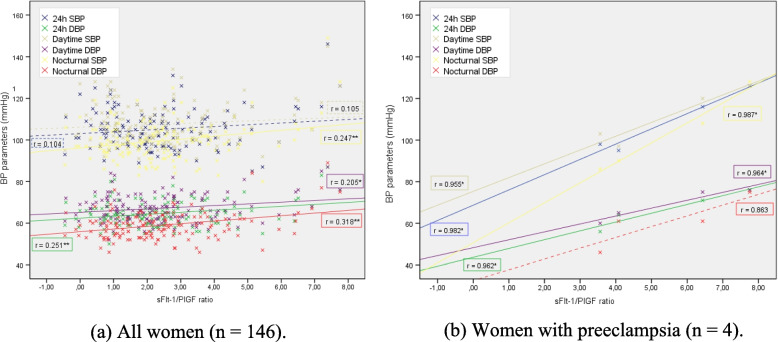
Correlation between ABPM and sFlt-1/PlGF ratio in whole population (**a**); in women with preeclampsia (**b**). * *p* < 0.05; ** *p* < 0.01. HDP: hypertensive disorders of pregnancy; PB: blood pressure; SBP: systolic blood pressure; DBP: diastolic blood pressure; sFlt-1/PlGF ratio: soluble fms-like tyrosine kinase-1 / placental growth factor ratio

Additionally, moderate negative correlations between PlGF levels and all ABPM parameters: systolic BP (24 h: *r* = − 0.301; daytime: *r* = − 0.289; and nocturnal: *r* = − 0.309; *p* < 0.001) and diastolic BP (24 h: *r* = − 0.353; daytime: *r* = − 0.356; and nocturnal: *r* = − 0.329; *p* < 0.001) were observed. Similarly, a low-moderate positive correlation was observed between the sFlt-1/PlGF ratio and the average 24 h diastolic BP (r: 0.251; *p* = 0.002), diastolic BP in the daytime period (r: 0.205; *p* = 0.013) and systolic (r: 0.247; *p* = 0.003) and diastolic BP (r: 0.318; *p* < 0.001) in the nocturnal period (Fig. 4a). When analysing these correlations separately in women who had preeclampsia (*n* = 4), stronger associations were found between ABPM parameters and PlGF levels for 24 h systolic BP (*r* = − 0.967; *p* = 0.033) and systolic BP in the daytime period (*r* = 0.973; *p* = 0.027), as well as the sFlt-1/PlGF ratio (Fig. 4b); however, these associations were not observed in women who presented with isolated gestational hypertension (without preeclampsia).

Table 4 summarizes the results of the final multivariate logistic regression model; following adjustment for potential confounding factors, sFlt-1/PlGF ratio (OR: 2.02), daytime SBP (OR: 1.27) and prepregnancy BMI (OR: 1.14) were found to be independent risk predictors for the development of HDP.

**Table 4:** Multivariate logistic regression for the risk prediction of HDP

Variable	OR	Z score	*p* Value	IC 95%
Age (y)	0.95	- 0.46	0.57	[0.81–1.11]
Prepregnancy BMI (kg/m^2^)	1.14	0.13	0.036	[1.01–1.30]
HbA1c (%)	0.96	- 0.03	0.88	[0.61–1.51]
Daytime SBP (mmHg)	1.27	0.11	0.036	[1.00–1.26]
Nocturnal SBP (mmHg)	0.92	- 0.07	0.21	[0.82–1.04]
sFlt-1/PIGF ratio	2.02	0.70	0.001	[1.35–3.05]

Multivariate analysis was performed using binary logistic regression models. The Hosmer–Lemeshow statistic (8.28 for 8 degrees of freedom (df); *p* = 0.41) indicates an adequate fit to the logistic regression model. *OR* odd ratio, *95% CI* 95% confidence interval, *BMI* body mass index, *HbA1c* glycated hemoglobin, *SBP* systolic blood pressure, *DBP* diastolic blood pressure, *sFlt-1/PlGF ratio* soluble fms-like tyrosine kinase-1 / placental growth factor ratio

## Discussion

Evidence exists that an angiogenic imbalance and increasing inflammatory biomarkers play a crucial role in both GDM and HDP [8, 20], as both entities share several pathophysiological features including endothelial dysfunction, oxidative stress and inflammatory activation. In the data we present here, we evaluated the association between the pro-inflammatory state and endothelial damage by measuring maternal serum inflammatory markers and the subclinical blood pressure alterations detected by ABPM during the third trimester of pregnancy in women with and without GDM.

The incidence of HDP in our study was 9.6%; of which, 78.6% (*n* = 11) presented with GDM and 57.1% (*n* = 8) with obesity. As described in the literature, maternal obesity is the most important modifiable risk factor for the development of HDP [25, 26] and GDM [27, 28]. In our cohort, the presence of obesity was similar in women with and without GDM; however, prepregnancy BMI was significantly higher in patients who developed HDP (Table 1). Although it has been previously described that increasing BMI is associated with a progressively increased risk of HDP [29–31], the mechanism by which excess adipose tissue causes the development of HDP in pregnant women remains unclear. Overweight is associated with alterations in lipid concentrations and the activation of inflammatory markers [32], and both of these metabolic abnormalities are characteristic of preeclamptic pregnancies before the onset of clinically evident disease [33]. In our study, we found higher levels of triglycerides in women with subclinical alterations in circadian rhythm, however, we could not establish its relationship in the pathogenesis of HDP, possibly due to the small number of patients developing HDP. Hence, in agreement with previous investigators, HDP may be secondary to an underlying placental insufficiency coupled with chronic oxidative stress from maternal metabolic disorders such as obesity and insulin resistance [30, 34]. In addition, we found that the rate of patients who received low doses of aspirin was higher in the HDP group, which we attributed to the fact that in these pregnant women presented more risk factors for preeclampsia and the indication of ASA prophylaxis.

On the other hand, GDM is also a recognized risk factor for developing HDP [5, 6, 35], although the rate is more variable [36] and could be influenced by glycemic control [37]. We observed higher basal glucose and HbA1c levels in women who subsequently presented with HDP, and in agreement with other studies, these showed a positive correlation between fasting glucose and HDP, even amongst women without GDM [38]. These results are consistent with the previous hypothesis that reported the causal role of insulin resistance in endothelial dysfunction and the development of HDP [8, 34]. Since insulin resistance can be indirectly measured by circulating adipokine markers, we investigated whether pro- and antiangiogenic biomarkers are related to subclinical BP alterations detected by ABPM in normotensive pregnant women who subsequently develop HDP.

It is widely recognized that there is an association between leptin concentration and fat mass, and since higher maternal leptin levels have been described in both pregnant women with obesity and pregnant women with GDM [12, 39] circulating leptin levels have been shown to be involved in the physiology of insulin resistance [40, 41]. Vitoratos et al. [42] found greater leptin levels in women with HDP, in agreement with a recent publication from our group among GDM women who develop HDP [13]. The data we present here demonstrate a low-moderate correlation between leptin levels and BP parameters when ABPM is performed before the development of HDP, and this correlation is even stronger amongt women who subsequently develop gestational hypertension or preeclampsia (Fig. 3).

With respect to other proinflammatory cytokines, a higher maternal concentration of MCP-1 was observed in women who developed HDP, as well as a positive correlation with BP levels, which is consistent with current studies describing that MCP-1 is involved in the endothelial inflammatory process [43], atherosclerosis and cardiovascular damage [44], and has also been described in preeclampsia [45].

In relation to angiogenic factors, evidence supports an angiogenic imbalance, characterized by greater concentrations of sFlt-1, lower PlGF levels and, particularly, a higher sFlt-1/PlGF ratio correlated with the risk of developing preeclampsia in pregnant women [9, 46–48] as well as those with preexisting diabetes [49, 50]. Recently, Nuzzo et al. [19] reported similar findings in women with GDM. Additionally, we have published a study describing that in a cohort of GDM women, the best predictor for HDP was the sFlt-1/PIGF ratio, which is directly correlated with diastolic BP values at delivery [13]. The relationship between BP levels and concentrations of sFlt-1, PlGF and sFlt-1/PlGF ratio has been investigated in women with chronic hypertension [51] and preeclampsia [46, 52, 53], but the association with BP values detected by ABPM has not been evaluated. Although in our cohort of patients the relationship between BP levels detected by ABPM and the sFlt-1/PlGF ratio was statistically significant, the correlation coefficient showed a low direct relationship, so any interpretation should be made with caution. Of note, the majority of studies, such as the SaPPPhirE Study [54], have measured cytokine markers in the development of preeclampsia or preeclampsia over preexisting hypertension, but did not include the development of gestational hypertension. It remains uncertain whether gestational hypertension and preeclampsia are different states of the same disease or two different entities [55]. Among previous studies that have also evaluated angiogenic biomarkers in other HDP, Yang et al. concluded that the sFlt-1/PlGF ratio is a valuable tool for the diagnosis of preeclampsia and severe preeclampsia rather than other types of HDP (including gestational hypertension and chronic hypertension) [56]. A similar conclusion was also reported by Engels et al. [57]. In our research, because of the small number of women who had HDP, we studied preeclampsia and gestational hypertension together, and it is possible that the lower incidence of events could influence the precision of our results. However, when we analysed the relationship between ABPM parameters and the concentration of PlGF and the sFlt-1/PlGF ratio in women who presented preeclampsia (*n* = 4), we observed a statistically significant stronger correlation, in line with the findings from the abovementioned studies about these angiogenic factors that may be useful in the prediction of preeclampsia compared to gestational hypertension.

Numerous studies consistently substantiate a strong association between an abnormal physiological circadian rhythm (including nondipper [58–60], extreme dipper [61] and riser patterns [62]) and a higher cardiovascular risk in hypertensive patients and in the general population [63]. The nondipper pattern has also been associated with cardiovascular events, left ventricular hypertrophy and a higher rate of clinical conditions, such as obstructive sleep apnea, diabetes or heart failure [64–66]. In pregnancy, several studies have attempted to determine whether ABPM could be a useful tool for predicting early changes in BP circadian rhythm in patients who subsequently develop HDP [17]. In fact, pregnant women with type 1 DM have shown an increase in the frequency of the nondipper pattern in the second trimester of pregnancy to be predictive of the development of HDP [67], and in previous studies of this cohort, our group reported that a predominance of nondipper patterns can be seen in GDM [68]. In our study, we found that the mean 24-h and daytime systolic and diastolic BP values were higher in women who subsequently developed HDP, and in agreement with previous studies [15], these patients had more frequently altered circadian patterns, although the results did not reach statistical significance. This altered circadian pattern was associated with higher levels of triglycerides and a higher sFlt-1/PlGF ratio, which could indicate that an atherogenic profile is related to a subclinical inflammatory status, the elevation of biomarkers and the alterations in BP circadian rhythm, all of which lead to vascular damage with implications for obstetric and perinatal outcomes.

To our knowledge, this is the first study to report the role of ABPM values as a valid predictor of HDP and to further evaluate inflammatory status and endothelial dysfunction using proinflammatory biomarkers and angiogenic factors in pregnant women who develop HDP. In our study, multivariate analysis identified daytime systolic BP and the sFlt-1/PlGF ratio as independent risk factors for the development of HDP. In addition, we found that prepregnancy obesity also increases the risk of developing HDP, and we have previously reported a positive correlation between ABPM parameters and prepregnancy BMI [18]. Therefore, we demonstrate that the use of ABPM along with the sFlt-1/PlGF ratio in women with GDM and obesity may be useful tools to identify those with an increased risk of developing HDP. This, in turn, could be of great value to the health system because a better prediction would entail a greater benefit both for the planning of prenatal care and for the adoption of effective strategies for the reduction of maternal and perinatal complications.

Nevertheless, there are several limitations in our study. First, 40% of participants (100 of 246 women enrolled) were excluded due to missing data which may result in potential selection bias. Second, the reproducibility of ABPM is limited, and there are few studies with which our results may be compared. Third, the small number of patients who developed HDP may have limited the power to detect some differences of smaller size, so our findings require confirmation in larger studies.

## Conclusions

In our cohort of normotensive pregnant women, HDP occurred significantly more frequently in women with GDM. BP values on ABPM are directly related to levels of leptin and MCP-1 as well as the sFlt-1/PlGF ratio, and inversely correlated with PlGF levels. We found that higher prepregnancy BMI, higher daytime systolic BP values on ABPM and an elevated sFlt-1/PlGF ratio were associated with an increased risk of developing HDP, and could be useful to identify women at higher risk who may benefit from further interventions to minimize the impact on pregnancy.

## Data Availability

The datasets used and/or analysed during the current study are available from the corresponding author on reasonable request.

## Abbreviations

ABPM: ambulatory blood pressure monitoring
AHT: arterial hypertension
ASA: acetysalicylic acid
BMI: body mass index
BP: blood pressure
DBP: diastolic blood pressure
FGF-2: fibroblast growth factor 2
GDM: gestational diabetes mellitus
HbA1c: glycated hemoglobin
HDL: high-density lipoprotein
HDP: hypertensive disorders of pregnancy
HGF: hepatocyte growth factor
IUGR: intrauterine growth restriction
LDL: low-density lipoprotein
LGA: large for gestational age
MCP-1: monocyte chemoattractant protein-1
NGF: nerve growth factor
ns: not statically significant
PAI-1: plasminogen activator inhibitor-1
PlGF: placental growth factor
SBP: systolic blood pressure
SD: standard deviation
sFlt-1: soluble fms-like tyrosine kinase-1
SGA: small for gestational age
TNF: tumor necrosis factor alpha
